# Supplementary material for: Ancestry Specific Polygenic Risk Score, Dietary Patterns, Physical Activity, and Cardiovascular Disease
Source: Nutrients. 2024 Feb 19;16(4):567. doi: 10.3390/nu16040567 (PMC10893526; doi:10.3390/nu16040567)
Supplement: Supplementary file 1 [file nutrients-16-00567-s001.zip › nutrients-2865349-supplementary.pdf]

Supplemental Materials

Table S1. Characteristics of diet forms used by the seven NHLBI Care studies.

Study	Diet Form	Items	Exam Year Used
ARIC	FFQ	semi-quantitative 66-item	1987-Exam 1
CARDIA	Diet history	102 questions	1989-Exam 1
CHS	Diet history	96 questions	1998-Exam 1
FHS OFFSPRING	FFQ	semi-quantitative 88-items	1983-Exam 3
FHS GENX 3	FFQ	semi-quantitative 88-items	2002-Exam 2
MESA	FFQ	semi-quantitative 153-items	2010-Exam 5
WHI	FFQ	semi-quantitative 145-items	2005-Exam 1

Abbreviations: NHLBI, National Heart, Lung, and Blood Institute; Care, Candidate Gene Association Resource; ARIC, Atherosclerosis Risk in Communities study,[28] CARDIA, Coronary Artery Risk Development in Young Adults Study,[32] CHS, Cardiovascular Heart Study,[33] FHS, Framingham Heart Study Offspring and GENX 3 studies,[30] MESA, Multi-Ethnic Study of Atherosclerosis Study,[29] and Women's Health Initiative study (WHI).[31]

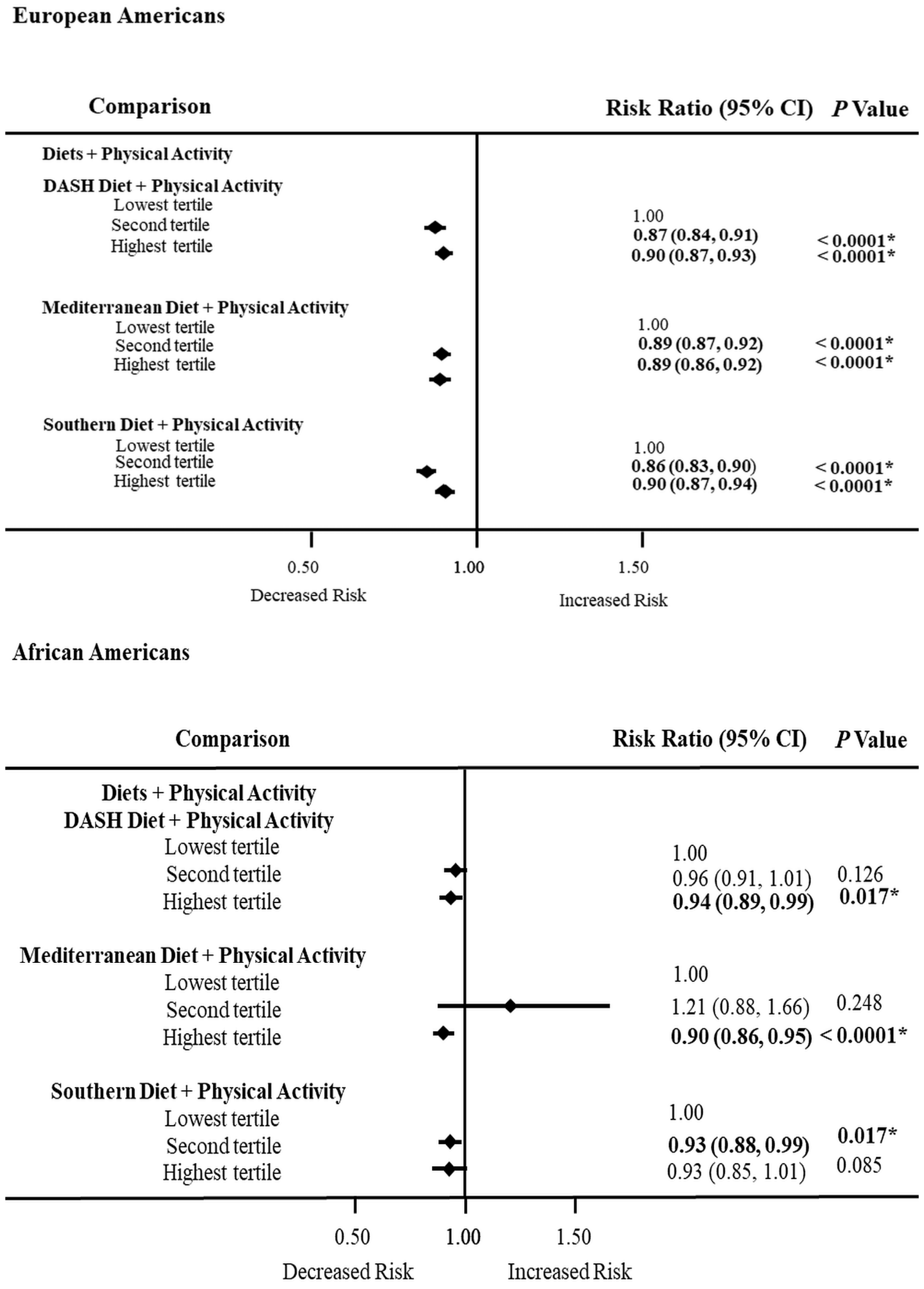

Figure S1. Association between dietary patterns and physical activity by ancestry.
Abbreviations: DASH, Dietary Approached to Stop High Blood Pressure.
Bold indicates *p* values that were statistically significant at $p < 0.05$.
*Bonferroni adjustment for multiple testing ($p < 0.05/2= < 0.025$).

PRS: Atherosclerotic Atherosclerosis cardiovascular disease status was regressed against an interaction term composed of each dietary scores and physical activity adjusting for a covariate propensity score composed of age, sex, current cigarette smoking status, and current drinking status.
Dietary pattern scores were computed using KMeans cluster, then converted to Z scores.
Physical activity was recoded as high/low by evaluating its functional form in each dataset.

Table S2. Genes shared between ancestries for European Americans and African Americans.

ABHD17AP4
ADAMTS7
BAK1
BCRP5
CCDC74BP1
CRKL
DPYSL2
EGFR
HLA-DPB2
LZTR1
PI4KA
PI4KAP2
POM121L4P
RPS18
SERPIND1
SMPD4P1
SNAP29
TMEM191A

Table S3. Top pathways of 18 overlapped genes between ancestries for European Americans and African Americans in the WikiPathway.

**Term**	***P* Value**	**Adjusted *P* Value**	**Genes**
22Q11 2 Copy Number Variation Syndrome WP4657	6.499^{-10}	5.459^{-8}	SERPIND1;PI4KA;LZTR1;SNAP29;EGFR;CRKL
Photodynamic Therapy Induced AP 1 Survival Signaling WP3611	9.134^{-4}	0.038	BAK1;EGFR
Endometrial Cancer WP4155	0.001	0.040	BAK1;EGFR
Non Small Cell Lung Cancer WP4255	0.002	0.040	BAK1;EGFR
Pancreatic Adenocarcinoma Pathway WP4263	0.003	0.041	BAK1;EGFR
ErbB Signaling Pathway WP673	0.003	0.041	EGFR;CRKL
TCA Cycle Nutrient Use And Invasiveness Of Ovarian Cancer WP2868	0.004	0.054	EGFR

Breast Cancer Pathway WP4262	0.008	0.082	BAK1;EGFR
EGF EGFR Signaling Pathway WP437	0.009	0.082	EGFR;CRKL

Adjusted *p* value used the Benjamini-Hochberg formula to adjust for false discovery rate.[51]
